# Strong Early Phase Parasympathetic Inhibition Followed by Sympathetic Withdrawal During Propofol Induction: Temporal Response Assessed by Wavelet-Based Spectral Analysis and Photoplethysmography

**DOI:** 10.3389/fphys.2021.705153

**Published:** 2021-09-13

**Authors:** Hsin-Yi Wang, Men-Tzung Lo, Kun-Hui Chen, Susan Mandell, Wen-Kuei Chang, Chen Lin, Chien-Kun Ting

**Affiliations:** ^1^Department of Anesthesiology, Taipei Veterans General Hospital, National Yang Ming Chiao Tung University, Taipei, Taiwan; ^2^Department of Biomedical Sciences and Engineering, National Central University, Taoyuan City, Taiwan; ^3^Department of Orthopedics and Traumatology, Taipei Veterans General Hospital, National Yang Ming Chiao Tung University, Taipei, Taiwan; ^4^Department of Anesthesiology, University of Colorado Hospital, Aurora, CO, United States

**Keywords:** propofol anesthesia, autonomic nervous system, heart rate variability, pulse photoplethysmography, wavelet-based spectral analysis

## Abstract

**Background:** Induction of anesthesia with propofol is associated with a disturbance in hemodynamics, in part due to its effects on parasympathetic and sympathetic tone. The impact of propofol on autonomic function is unclear. In this study, we investigated in detail the changes in the cardiac autonomic nervous system (ANS) and peripheral sympathetic outflow that occur during the induction of anesthesia.

**Methods:** Electrocardiography and pulse photoplethysmography (PPG) signals were recorded and analyzed from 30 s before to 120 s after propofol induction. The spectrogram was derived by continuous wavelet transform with the power of instantaneous high-frequency (HFi) and low-frequency (LFi) bands extracted at 1-s intervals. The wavelet-based parameters were then divided into the following segments: (1) baseline (30 s before administration of propofol), (2) early phase (first minute after administration of propofol), and (3) late phase (second minute after administration of propofol) and compared with the same time intervals of the Fourier-based spectrum [high-frequency (HF) and low-frequency (LF) bands]. Time-dependent effects were explored using fractional polynomials and repeated-measures analysis of variance.

**Results:** Administration of propofol resulted in reductions in HFi and LFi and increases in the LFi/HFi ratio and PPG amplitude, which had a significant non-linear relationship. Significant between-group differences were found in the HFi, LFi, and LFi/HFi ratio and Fourier-based HF and LF after dividing the segments into baseline and early/late phases. On *post hoc* analysis, changes in HFi, LFi, and the LFi/HFi ratio were significant starting from the early phase. The corresponding effect size (partial eta squared) was > 0.3, achieving power over 90%; however, significant decreases in HF and LF were observed only in the late phase. The PPG amplitude was increased significantly in both the early and late phases.

**Conclusion:** Propofol induction results in significant immediate changes in ANS activity that include temporally relative elevation of cardiac sympathovagal balance and reduced sympathetic activity.

**Clinical Trial Registration:** The study was approved by the Institutional Review Board of Taipei Veterans General Hospital (No. 2017-07-009CC) and is registered at ClinicalTrials.gov (https://clinicaltrials.gov/ct2/show/NCT03613961).

## Introduction

Propofol belongs to a group of alkylphenols whose hypnotic action is mediated by enhancing γ-aminobutyric acid (GABA)-induced chloride current through its binding to the β subunit of the GABAA receptor. It is one of the most commonly used induction anesthetics. However, administration of propofol for induction of anesthesia can cause changes in heart rate and hypotension, requiring treatment to maintain hemodynamic stability (Hermann and Vettermann, 1992; Burjorjee and Milne, 2002; Douglas and Cadogan, 2008). The decision as to when and how to treat hemodynamic changes remains empiric because of our incomplete understanding of propofol’s physiological effects on the cardiovascular system. Recent studies suggest that propofol affects overall activity and balance in the autonomic nervous system (ANS) (Hermann and Vettermann, 1992; Burjorjee and Milne, 2002; Douglas and Cadogan, 2008). However, there is conflicting evidence regarding the influence of propofol on ANS activity (Ebert et al., 1992; Scheffer et al., 1993; Deutschman et al., 1994; Kanaya et al., 2003). There is evidence from spectral analysis of heart rate variability (HRV) of a correlation between the integrity and balance of sympathetic and parasympathetic activation (Eckberg et al., 1985; Malliani et al., 1991). Two common spectral analyses of power frequencies embedded within the cardiac beat-to-beat (R-R) interval on electrocardiography (ECG) have been correlated with components of the ANS. The spectral analysis-derived high frequency (HF, 0.15−0.4 Hz) power of a heartbeat series has been used as a marker of parasympathetic modulation (Malliani et al., 1991) while low frequency (LF, 0.04−0.15 Hz) power, which is synchronous with vasomotor waves, is considered to be mediated by the parasympathetic and sympathetic systems (Akselrod et al., 1981; Galletly et al., 1994; Kanaya et al., 2003). The LF/HF ratio has been used as an indicator of the balance between sympathetic and vagal modulation (Akselrod et al., 1981; Montano et al., 1994). However, the effects of propofol on the functioning of the ANS vary according to the measurement methods used (Scheffer et al., 1993; Deutschman et al., 1994; Galletly et al., 1994). Deutschman et al. demonstrated that propofol bolus anesthesia reduces parasympathetic tone to a lesser degree than sympathetic tone, resulting in a parasympathetic dominant response for bradycardia (Burjorjee and Milne, 2002). However, Galletly et al. (1994) stated that propofol resulted in a greater reduction in HF power than LF power, indicating that propofol anesthesia reduces parasympathetic tone greater than sympathetic tone, suggesting sympathetic dominance. Similar sympathetic-dominance results were observed after induction of anesthesia with propofol (Hermann and Vettermann, 1992; Douglas and Cadogan, 2008). Methods commonly used to assess cardiac ANS activity, such as Fourier-based spectral analysis (FBSA) (Kanaya et al., 2003), can lose information that could be physiologically important. Averaging electrical conduction impulses and the stationary assumption in FBSA would result in the relative dampening of the integrity and balance of sympathetic and parasympathetic activity; therefore, this method is unable to detect temporal changes in cardiac ANS activity, which limits its application during induction of anesthesia (Ebert et al., 1992; Ebert and Muzi, 1994; Guenoun et al., 2000).

To overcome these limitations, we have used wavelet-based spectral analysis (WBSA), which fits signals to multiple wavelets, captures more detail, and improves time resolution. Another potential source of conflicting data may relate to differences in the magnitude of drug effects. Therefore, we analyzed HRV in a granular fashion on a per-second basis to capture propofol’s effect during the induction period. We have also improved the separation between parasympathetic and sympathetic activity by incorporating pulse photoplethysmography (PPG) signals (Ahonen et al., 2007; Hamunen et al., 2012).

This study aimed to better characterize the changes that occur in the ANS following a bolus dose of propofol to understand how the ANS could influence hemodynamic stability following induction of anesthesia.

## Materials and Methods

This open prospective observational study included 30 patients with American Society of Anesthesiologists physical status I–II who underwent elective surgery and induction of general anesthesia with propofol between July 2017 and July 2018. The study was approved by the Institutional Review Board of Taipei Veterans General Hospital (IRB No. 2017-07-009CC) and registered at ClinicalTrials.gov (NCT03613961). Written informed consent was obtained from all study participants. The manuscript adheres to all applicable STROBE guidelines.

Patients who had recently taken a sedative, beta-blocker, parasympatholytic, or opioid agent were excluded, as were those who had undergone emergency surgery and those with a history of hypovolemia, hypothermia, arrhythmia, diabetes, hypertension, or impaired renal, hepatic, cardiac, or respiratory function, any of which could interfere with the effects of propofol on the regulation of ANS activity. Patient age, weight, and height were recorded (see Supplementary Table 1).

Standard monitoring (ECG, pulse oximetry, and blood pressure monitors) was used, and recordings were made before and throughout induction of anesthesia. ECG waveforms were continuously recorded using a multichannel polygraph system (Embla N7000, Natus, Pleasanton, CA, United States). The data were saved at a rate of 1,024 Hz directly to a memory card in the device for offline analysis of HRV and PPG. All data included in the analysis of the immediate effects of propofol were obtained from continuous artifact-free ECG recordings. All patients received 100% oxygen via a face mask for 2–3 min before induction, and recordings were obtained while the patient was lying quietly in a supine position and breathing spontaneously. During the study sequence, the patient was asked to lie calmly and breathe in rhythm with a metronome at a rate of 10 breaths/min (0.167 Hz). The patient was requested not to talk and was pre-treated with lidocaine 0.2 mg/kg to minimize any pain at the injection site. All patients were induced with propofol 1.5–2.5 mg/kg, and the sequences from 30 s before to 2 min after administration were selected for analysis. Each patient received propofol (Propofol-Lipuro 1%, B Braun, Melsungen, Germany) via a 20-gauge catheter [sited in a forearm vein opposite to that used for measurement of blood pressure (BP), to avoid interruption of propofol administration] followed by manual ventilation with a tidal volume of 6 ml/kg and a respiratory rate of 10/min with 100% oxygen as required via a mask to maintain normal ventilation. External stimulation was minimized and included only the basic maneuvers required to maintain a patent airway. The study protocol and general anesthesia were then carried out and surgery was performed in the usual manner.

### Processing Analysis

#### Pre-processing of ECG Data

The R waves were initially detected from the ECG recording, visually validated, and corrected to ensure accurate detection of normal heartbeats. The corrected R-R intervals in the time series were resampled by cubic spline interpolation at an even interval of 0.125 of a second to meet the prerequisites for wavelet and Fourier analysis (see section “Introduction,” Supplementary Text 1, describing the methods used to process the ECG data).

#### Wavelet and Fourier Analysis of R-R Intervals

HF and LF power were calculated at 1-s intervals with continuous wavelet function, and the instantaneous HF (HFi), LF (LFi) components, and the LFi/HFi ratio were derived, representing the instantaneous function of the ANS (see section “Materials and Methods,” Supplementary Text 1). In contrast, the Fourier spectrum power over the HF and LF band was calculated according to the published guidelines (Task Force of the European Society of Cardiology and the North American Society of Pacing and Electrophysiology, 1996), with a time interval of 1 min after administration of propofol (see section “Results,” Supplementary Text 1). The HF of the series of heartbeats was used as a marker of vagal modulation (Pomeranz et al., 1985) and the ratio of the LF to the HF (LF/HF) was used as an indicator of the balance between sympathetic and vagal modulation (Malliani et al., 1991; Montano et al., 1994).

#### Beat-to-Beat Pulse Amplitudes From Photoplethysmography

The amplitude between the trough and peak of each pulse wave on PPG was calculated to extract the PPG amplitude (PPGA; see section “Discussion,” Supplementary Text 1). The peripheral sympathetic response, including the decrease in PPGA associated with sympathetic vasoconstriction after induction with propofol, can be detected by PPG (Babchenko et al., 2001; Colombo et al., 2015).

### Statistical Analysis

The HRV derived from the wavelet-based and PPGA values after administration of propofol were analyzed using fractional polynomials at a series of time points to preserve both the continuous and dynamic results of WBSA. The goodness-of-fit was assessed during the building of the model. The continuous readings were divided into three discrete time windows: (1) baseline (30 s prior to propofol administration), (2) early phase (1-min following propofol administration), and (3) late phase (the second minute following propofol induction). A one-way repeated-measures analysis of variance with *post hoc* comparisons (Sidak correction) was then performed to compare the results for parameters derived from WBSA and FBSA in the three phases of induction.

The primary study outcomes were early changes in the sympathetic and parasympathetic nervous systems and the sympathovagal balance in the heart in response to a bolus of propofol as detected by WBSA. The normality test for the data were checked during the statistical model building. Previous studies assumed that a 10% difference in HRV parameters relative to baseline would be relevant (Kanaya et al., 2003; Win et al., 2005; El Beheiry and Mak, 2013). In this study, an average change in R-R interval of −56.2 ± 106.2 ms was noted when the LFi/HFi ratio met the 10% threshold change from baseline after administration of propofol. The sample size calculation was based on an F test with repeated-measures analysis of variance within factors, an effect size of 0.3, a power of 85%, and a significance level of 5%. Allowing for missing data, artifacts, and technical problems precluding analysis of HRV and PPG in 25% of subjects, it was determined that 30 subjects would need to be enrolled to ensure a final sample size of 22. The data are expressed as the mean ± standard deviation of mean. All analyses were performed using STATA 12 (Stata Corporation, College Station, TX, United States). A *P*-value < 0.05 was considered statistically significant.

## Results

**FIGURE 1:**
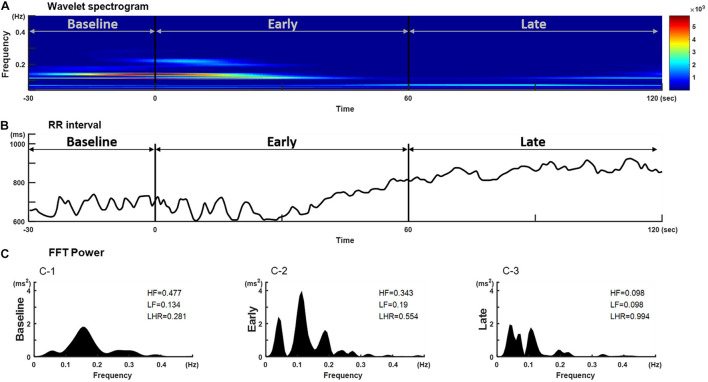
Temporal changes in **(B)** R-R interval, **(A)** its corresponding wavelet-based spectrogram, and **(C)** the Fourier spectrum in a representative individual after a bolus of propofol. The wavelet-based spectrogram in **(A)** shows the change in the frequency content of signals over time and a decrease in HFi and LFi power in response to propofol at the start of induction. Frequency is plotted on the y-axis and time on the x-axis. The amount of energy or power in the signal is indicated in color. The spectrogram shows the power content in the range of 0.01–0.40 Hz. **(C)** The corresponding RR interval signal obtained during the different phases after induction of propofol. HF and LF power derived by the traditional Fourier-based method shows decreases only in the late phase after induction. HF, high-frequency power; HFi, instantaneous high-frequency power; LF, low-frequency power; LFi, instantaneous low-frequency power.

**FIGURE 2:**
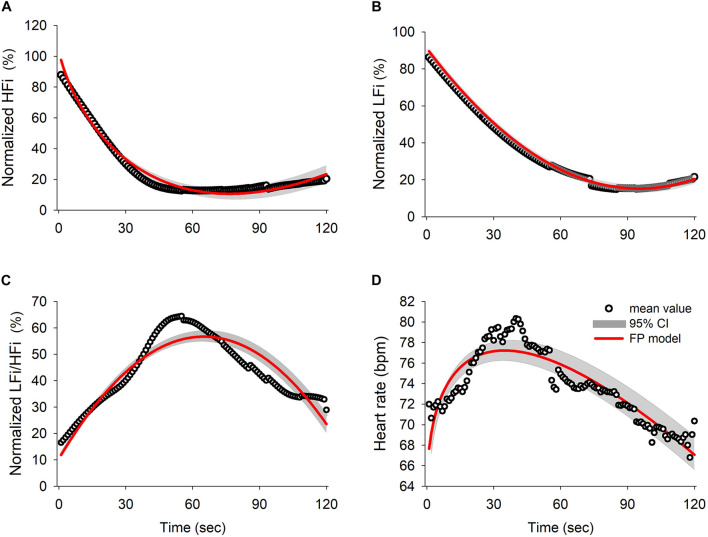
Results of the fractional polynomials with 95% confidence interval and goodness-of-fit in **(A)** normalized HFi (R squared = 0.9828), **(B)** normalized LFi (R squared = 0.999), **(C)** normalized LFi/HFi ratio (R squared = 0.8635), and **(D)** heart rate (R squared = 0.8273). The black circles represent the averaged value for each derived parameter for all subjects over time and the red line illustrates the fitted result for the fractional polynomial model with the 95% confidence interval (shadowed area). HFi, instantaneous high-frequency power; LFi, instantaneous low-frequency power.

**FIGURE 3:**
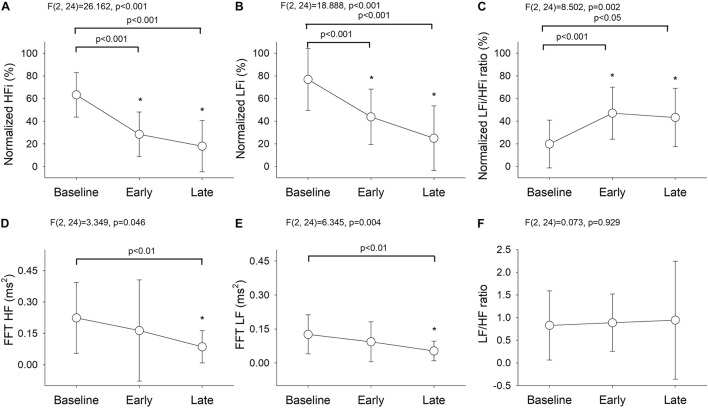
Temporal changes in **(A)** normalized HFi, **(B)** normalized LFi, **(C)** normalized LFi/HFi ratio, **(D)** Fourier-based HF, **(E)** Fourier-based LF, and **(F)** Fourier-based LF/HF ratio at baseline and in the early and late phases. The main effect was assessed by repeated-measures analysis of variance. Baseline phase, 30 seconds before induction; early phase, the first minute of propofol induction; late phase, the second minute of propofol induction. The data are presented as the mean ± the standard error of the mean. *P < 0.05 vs. baseline, *post hoc* test. ANOVA, analysis of variance; HF, high-frequency power; HFi, instantaneous high-frequency power; LF, low-frequency power; LFi, instantaneous low-frequency power.

Thirty eligible patients consented to participate in the study. Data of five subjects were excluded from the analysis because of movement-related artifacts. The patient characteristics are summarized in Supplementary Table 1. The mean ± standard error of mean of patients’ age was 44.8 ± 2.67 years and 56% of the patients were women. The mean weight and height were 160.50 ± 1.43 cm and 62.28 ± 1.87 kg, respectively, and the mean body mass index (24.10 ± 0.63) was within the normal range. Figure 1 shows a representative tachogram for the R-R intervals, including a typical WBSA (Figure 1A) of the R-R intervals (Figure 1B) and the FBSA of the different induction phases (Figure 1C). Temporal increases in HFi and LFi were noted between the end of the baseline period and the start of administration of propofol, when the patient is supposed to be in a relaxed state. Subsequently, there was a decrease in HFi and LFi power in response to propofol in the middle of the early phase. In contrast, values for HF or LF power derived by the traditional method were equivocal in the early phase (Figures 1C–2) but were markedly decreased in the late phase (Figures 1C–3). A rapid decrease in HFi and LFi power in response to propofol was seen in the early phase (Figure 1A, middle). Therefore, we used a fractional polynomial analysis to evaluate wavelet-derived dynamic ANS activity (LFi, HFi, and the LFi/HFi ratio) and changes in heart rate (HR) after induction and found a significant non-linear relationship (Figure 2 and Supplementary Table 2). The averaged normalized HFi, LFi, and LFi/HFi ratio and HR of all study participants at the same time points after administration of propofol and the curve fitted from the derived fractional polynomials (red line) are shown in Figure 2. A decrease in LFi and HFi power was noted in the early phase after administration of propofol (Figures 2A,B); however, the LFi/HFi ratio increased at first in response to a greater decline in HFi and decreased thereafter (Figure 2C). Like the LFi/HFi ratio, the HR increased in the early phase and then decreased (Figure 2D).

Division of the data into three phases revealed significant between-group differences in the results obtained using the wavelet-based method {HFi [*F*(2, 24) = 26.162, *P* < 0.001], LFi [*F*(2, 24) = 18.888, *P* < 0.001], and LFi/HFi ratio [*F*(2, 24) = 8.502, *P* = 0.002]; Figures 3A–C} and the Fourier-based method {HF [*F*(2, 24) = 3.349, *P* = 0.046] and LF [*F*(2, 24) = 6.345, *P* = 0.004]; Figures 3D,E}. There were no significant changes in the LF/HF ratio derived by FBSA after the bolus of propofol [*F*(2,24) = 0.073, *P* = 0.929; Figure 3F]. The *post hoc* test showed that the decrease in HFi and LFi values and the increase in the LFi/HFi ratio were most significant between baseline and the early phase and between baseline and the late phase but that the changes in the Fourier-based HF and LF were only significant between baseline and the late phase (Figure 3). Comparison of the LFi/HFi ratio from the average data (Figure 3C) and higher resolution (Figure 2C) by wavelet-based analysis suggested that the early and late phases were identical with some flattening of the relationship after induction when the average data was used.

**FIGURE 4:**
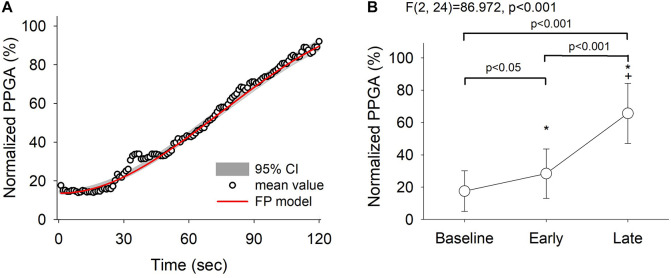
Fractional polynomials with 95% confidence interval and goodness-of-fit of normalized pulse photoplethysmography amplitude (PPGA) changes (R squared = 0.992) **(A)** and the corresponding PPGA assessed by repeated-measures analysis of variance **(B)**. Baseline, 30 s before induction; early phase, the first minute of propofol induction; late phase, the second minute of propofol induction. The data are presented as the mean ± the standard error of the mean. *P < 0.05 vs. baseline, post hoc test; ^†^*P* < 0.05 vs. early phase, *post hoc* test. A continued dilatation response of PPGA was clearly demonstrated. ANOVA, analysis of variance.

The averaged PPGA with the curve fit from the derived fractional polynomials (the red line in Figure 4A) showed that the mean PPGA varied with time (see Supplementary Table 2). A continuous sympathetic inhibition response was clearly demonstrated. Furthermore, a *post hoc* test showed that the increase in PPGA was most marked between the baseline/early and late phases [Figure 4B, *F*(2, 24) = 86.972, *p* < 0.001]. These findings indicate that there was an overall early reduction in the autonomic response after administration of propofol; which was observed as an early reduction in HFi and LFi (Figure 2) and a continuous increase in normalized PPGA (Figure 4).

## Discussion

Our findings showed that induction with propofol causes changes in the ANS that can be divided into two phases. Although there was an overall reduction in ANS activity, WBSA identified a relative sympathetic activity enhancement at the cardiac ANS level early after induction that subsided as time progressed. In contrast, measurements obtained by FBSA failed to identify these changes. The averaged HRV data produced by FBSA likely failed to capture important biphasic changes detected only when using WBSA. The disparity in findings between FBSA and WBSA likely explains the conflicting literature regarding the effects of propofol on ANS activity. Overall, after propofol administration, we found that a reduction in total autonomic activity manifested as a significant decrease in HFi and LFi power and an increase in PPGA. Using WBSA, we could detect the time course of these changes. The first change is a significant early reduction in HFi power, followed by a reduction in LFi power, which leads to a shift in the balance of ANS function toward relative sympathetic dominance at the start of induction. The significant early reduction in HFi power after a propofol bolus achieved relatively stronger parasympathetic inhibition and shifted the balance of ANS function toward a more sympathetic dominance (an elevated LFi/HFi ratio and HR). In the late phase, the sympatholytic effect became prominent, as indicated by a greater increase in PPGA.

### Use of Wavelet-Based Spectral Analysis to Extract the Dynamics of ANS Activity During Induction of Anesthesia

Autonomic abnormalities may lead to cardiac dysfunction and increase the possibility of hemodynamic instability (Zhang et al., 2012). Furthermore, sympathetic overactivity has an important role in the generation of arrhythmias (Douglas and Cadogan, 2008), and the inhibitory effect of parasympathetic impulses is important in ventricular arrhythmias related to acute myocardial ischemia (Meesmann et al., 1991). Propofol is widely used as an induction agent due to the rapid onset of anesthesia after bolus administration. However, severe cardiac events related to propofol induced-autonomic dysfunction have also been reported (Guise, 1991). We believe that the most disturbance occurs during induction of anesthesia using propofol (Hermann and Vettermann, 1992; Burjorjee and Milne, 2002; Douglas and Cadogan, 2008) and that higher-resolution monitors are needed to identify exactly how propofol modifies the activity of the cardiac ANS. This would be important for avoiding adverse consequences and allowing more prompt diagnosis and treatment. We found propofol bolus-related parasympathetic withdrawal with relative sympathetic enhancement at the cardiac ANS level in the first minutes after administration of the bolus and an overall decrease in sympathetic activity. The initial increase in HR could be interpreted as an early change in the sympathovagal balance toward predominantly sympathetic modulation of the cardiac ANS. A non-linear method that can measure HRV may enhance the monitoring of sympathetic and parasympathetic influences, especially in this unstable stage of anesthesia (Oida et al., 1997). Furthermore, we compared averaged data for the first and second minutes with those obtained by FBSA. The dynamics of the initial early increase and subsequent decrease in sympathovagal balance could not be detected in detail by the average data, even with wavelet-based analysis. Using average data would lead to the loss of important clinical information. Moreover, conventional FBSA is insensitive to temporal changes in cardiac ANS activity because of the stationary assumption and use of average data, especially during induction of anesthesia (Ebert et al., 1992; Ebert and Muzi, 1994; Guenoun et al., 2000).

### Relationship Between Instantaneous Cardiac ANS Activity and PPG-Derived Peripheral Sympathetic Parameters

Our findings showed parasympathetic withdrawal with sympathovagal balance shift toward relative sympathetic enhancement at the cardiac ANS level in the early phase after a propofol bolus and a steady decrease in sympathetic vascular activity.

Several studies have explored the effect of propofol on peripheral vascular circulation. Robinson et al. (1997) found that infusion of propofol into the brachial artery did not produce a significant vascular response despite therapeutic plasma concentrations in the forearm. The effects of propofol on venous compliance and resistance in the forearm were similar to the effects of sympathetic denervation by stellate ganglion block. The mechanism of propofol-mediated arterial and venous dilation appears to be more likely due to inhibition of sympathetic vasoconstrictor nerve activity (Ebert et al., 1992; Sellgren et al., 1994) than due to direct vascular relaxation (Robinson et al., 1997). Accordingly, central mechanisms would be involved in the initial vasodilatation observed during propofol anesthesia (Neukirchen and Kienbaum, 2008). Sympathetic vasoconstrictor outflow to the blood vessels can be directly investigated by sympathetic microneurography, which assesses regional sympathetic activity during anesthesia, but this is difficult to perform in clinical practice. PPG has been shown to reflect changes in vasoconstrictor nerve activity in the skin (Hamunen et al., 2012). An increase in arterial compliance can manifest as an increase in PPGA during general anesthesia and could be a convenient and non-invasive clinical assessment method in physiological and pharmacological studies. In this study, we analyzed PPGA and found durable suppression of sympathetic activity after administration of propofol. Finger blood flow is regulated mainly by local arteriovenous shunts (Sessler et al., 1992), and we speculate that the prolonged sympatholytic response, rather than the HRV response, may be in part due to reduced release of sympathetic neurotransmitters. This theory suggests an association between sympathectomy-induced peripheral vasodilation and increased PPGA, as reported previously (Babchenko et al., 2001; Colombo et al., 2015). Most investigators agree that FBSA of HRV shows that propofol suppresses parasympathetic tone; however, its effect on sympathetic tone is controversial (Ebert et al., 1992; Scheffer et al., 1993; Ebert and Muzi, 1994; Guenoun et al., 2000; Kanaya et al., 2003). HRV determination may be suitable for assessment of the parasympathetic influences on HR but does not reflect cardiac sympathetic innervation (Neukirchen and Kienbaum, 2008). Our laboratory has chosen a model that separates sympathetic and parasympathetic nerve stimuli more reliably and calculates the resolution per second rather than using averaged data. WBSA was used to investigate the balance response of the cardiac ANS and incorporated PPG signals, which are a sensitive marker of sympathetic activity in anesthetized patients (Ahonen et al., 2007; Hamunen et al., 2012), to obtain information on changes in peripheral sympathetic control. This method is non-invasive and could be used in the clinical setting.

We attributed the initial acceleration of HR in our study to the parasympatholytic effect of propofol on the predominant parasympathetic activity during the resting baseline status, which caused a greater reduction in the HFi than in the LFi in the early phase after propofol administration, with a shift in the ratio toward sympathetic dominance. Moreover, the LFi/HFi ratio started to increase in this early phase, which also tipped the balance toward sympathetic dominance. The transient increase in HR in the early induction phase can be interpreted as an early change in the sympathovagal balance toward predominantly sympathetic modulation of the cardiac ANS. Propofol has been shown to decrease peripheral vascular resistance in humans via direct inhibition of sympathetic vasoconstrictor nerve activity (Robinson et al., 1997) rather than via compensatory changes in the ANS related to peripheral vasodilation. Using sympathetic microneurography, Ebert et al. (1992) examined the HR response to hypotension and demonstrated that propofol reduced tonic sympathetic nerve activity and markedly attenuated the baroreflex changes in sympathetic activity in response to changes in BP. They concluded that the sympathoinhibitory effect of propofol contributed to the subsequent hypotension (Ebert et al., 1992; Guyenet, 2006). In our study, we used non-invasive PPG, which detects changes in skin vasoconstrictor nerve activity and found an increase in PPGA in the early and late phases after administration of propofol, which could be explained by a direct sympatholytic effect leading to vasodilation. It would be interesting to understand the subsequent relationship between lowering of arterial BP and resetting of the baroreflex operating point to restore the preset BP level (Ebert et al., 1992) after induction of anesthesia with propofol.

The main limitation of this study is that propofol-induced changes in respiratory rate and tidal volume may have influenced the HF power of the R-R intervals (Hayano et al., 1994). We addressed this issue by minimizing external stimulation and instructing the patients to lie down calmly and maintain a steady respiration before induction of anesthesia by following a metronome set at a respiratory rate of 10/min. The temporal increase in HFi is proof of relaxation before induction. We maintained this ventilation rate after administration of propofol to minimize the influence of respiratory rate in the measurement propofol-related HRV changes. Moreover, WBSA fitted to the signal with multiple wavelets and the temporal changes of the signal can be assessed with higher time resolution (e.g., the amplitude and frequency of each wavelet) (Gans et al., 2009). However, a wavelet-based second-by-second higher time resolution approach would overestimate the relationship with nervous system activity, and we did not confirm the autonomic function by direct measurement. Further validation studies during propofol induction may be needed. Furthermore, we excluded high-risk patients, including those with diabetes and the elderly. How these conditions affect the activity of the ANS and alter its responses to propofol remains unclear. Further research is needed to evaluate the relevance of these variables under various anesthetic and surgical conditions across different patient groups.

## Conclusion

In conclusion, a combination of PPG and WBSA may provide a more comprehensive measure of the temporal cardiovascular ANS modulation by propofol and be useful for detecting changes in ANS activity. Awareness of these data will provide a stronger scientific basis for the early identification of abnormal physiological responses to propofol and allow appropriate treatment strategies.

## Data Availability Statement

The raw data supporting the conclusions of this article will be made available by the authors, without undue reservation.

## Ethics Statement

The studies involving human participants were reviewed and approved by the Institutional Review Board of Taipei Veterans General Hospital (No. 2017-07-009CC). The patients/participants provided their written informed consent to participate in this study.

## Author Contributions

H-YW helped to develop the protocol, obtain data for analysis, and draft the article. CL and M-TL helped to extract and analyze the data. K-HC helped to obtain data for analysis. SM helped to design the protocol and interpret the results of the analysis. W-KC helped to interpret the results of the analysis and participated in the final review of the manuscript. C-KT helped to develop the protocol, interpret the results of the analysis, and participated in the final review of the manuscript. All authors contributed to the article and approved the submitted version.

## Conflict of Interest

The authors declare that the research was conducted in the absence of any commercial or financial relationships that could be construed as a potential conflict of interest.

## Publisher’s Note

All claims expressed in this article are solely those of the authors and do not necessarily represent those of their affiliated organizations, or those of the publisher, the editors and the reviewers. Any product that may be evaluated in this article, or claim that may be made by its manufacturer, is not guaranteed or endorsed by the publisher.

## Abbreviations

PPG: pulse photoplethysmography
HFi: instantaneous high-frequency
LFi: instantaneous low-frequency
HF: high-frequency
LF: low-frequency
ANS: autonomic nervous system
HRV: heart rate variability
R-R: beat-to-beat
ECG: electrocardiography
FBSA: Fourier-based spectral analysis
WBSA: wavelet-based spectral analysis
BP: blood pressure
PPGA: PPG amplitude.
