# Supplementary material for: Strong Early Phase Parasympathetic Inhibition Followed by Sympathetic Withdrawal During Propofol Induction: Temporal Response Assessed by Wavelet-Based Spectral Analysis and Photoplethysmography
Source: Front Physiol. 2021 Sep 13;12:705153. doi: 10.3389/fphys.2021.705153 (PMC8473792; doi:10.3389/fphys.2021.705153)
Supplement: Supplementary file 1 [file Data_Sheet_1.docx]

Supplementary Material

# Supplementary tables

**Supplemental Table S1** Demographic and anthropometric characteristics of the study participants

| Age, years |  | 44.8 ± 2.67 |  |  |  |
| --- | --- | --- | --- | --- | --- |
| Sex |  |  |  |  |  |
| Female |  | 14 (56%) |  |  |  |
| Male |  | 11 (44%) |  |  |  |
| BMI |  | 24.10 ± 0.63 |  |  |  |
| Height, cm |  | 160.50 ± 1.43 |  |  |  |
| Bodyweight, kg |  | 62.28 ± 1.87 |  |  |  |
|  |  |  |  |  |  |
|  | | | | | |

The data are shown as the mean ± standard error of mean or as the number (percentage). BMI, body mass index (weight in kilograms divided by the square of the height in meters).

**Supplemental Table S2.** Results of second-degree fractional polynomials

|  | Coefficient | S.E. | p | Deviance | Deviance difference | p (*) |
| --- | --- | --- | --- | --- | --- | --- |
| Normalized HFi |  |  |  | 14721.343 | 6396.326 | <0.001 |
| Intercept | 104.164 | 0.207 | <0.001 |  |  |  |
| Time | −6.517 | 0.022 | <0.001 |  |  |  |
| Time*ln(Time) | 1.221 | 0.004 | <0.001 |  |  |  |
| Normalized LFi |  |  |  | 6283.756 | 12980.822 | <0.001 |
| Intercept | 89.558 | 0.038 | <0.001 |  |  |  |
| Time | −1.580 | 0.001 | <0.001 |  |  |  |
| Time^2^ | 0.008 | 0.000 | <0.001 |  |  |  |
| Normalized LFi /HFi |  |  |  | 18116.954 | 4993.789 | <0.001 |
| Intercept | 11.862 | 0.268 | <0.001 |  |  |  |
| Time | 1.401 | 0.010 | <0.001 |  |  |  |
| Time^2^ | −0.011 | 0.000 | <0.001 |  |  |  |
| HR |  |  |  | 10718.812 | 2571.755 | <0.001 |
| Intercept | 63.349 | 0.165 | <0.001 |  |  |  |
| Time^0.5^ | 4.701 | 0.052 | <0.001 |  |  |  |
| Time | −0.398 | 0.004 | <0.001 |  |  |  |
| Normalized PPGA |  |  |  | 13492.334 | 2910.162 | <0.001 |
| Intercept | 13.637 | 0.082 | <0.001 |  |  |  |
| Time^2^ | 0.011 | 0.000 | <0.001 |  |  |  |
| Time^3^ | 0.000 | 0.000 | <0.001 |  |  |  |

The best-fitting second degree fractional polynomials are shown, and a statistical testing against the best-fitting first degree fractional polynomials through deviances was performed. (*) p-value from deviance difference comparing first-degree with second-degree fractional polynomial. HF, high-frequency power; HFi, instantaneous high-frequency power; HR, heart rate; LF, low-frequency power; LFi, instantaneous low-frequency power; PGGA, photoplethysmography amplitude

# Supplemental Text 1

## Section 1: Pre-processing of ECG data

In this study, the R waves were first detected using the automatic algorithm with a detection accuracy of ~99.98% based on the online MIT-BIH Arrhythmia Database (<https://physionet.org/>). A bandpass finite-impulse response filter was applied on the ECG signal to remove baseline wandering and increase the accuracy of the QRS detector even in the presence of a noisy signal. Furthermore, the algorithm automatically identifies abnormal beats, such as premature atrial and ventricular contractions. Next, the QRS waves were detected using a temporally adaptive threshold to find the locally prominent activation wave (R wave).^1^ Supplemental Figure S1 illustrates the QRS detection procedure for the recorded ECG data. The raw data (A) were bandpass-filtered, and an adaptive QRS detector was then applied to the data (B). The automated annotated results generated by the QRS detector were visually inspected and corrected (C). Finally, normal-to-normal sinus intervals, extracted from the corrected R-R interval time series (D; black line) by removing the abnormal beats, were resampled and interpolated by cubic spline interpolation at an even interval of 0.125 of a second (i.e., 8 Hz; D, red line). These steps ensure high-quality detection of normal heartbeats.


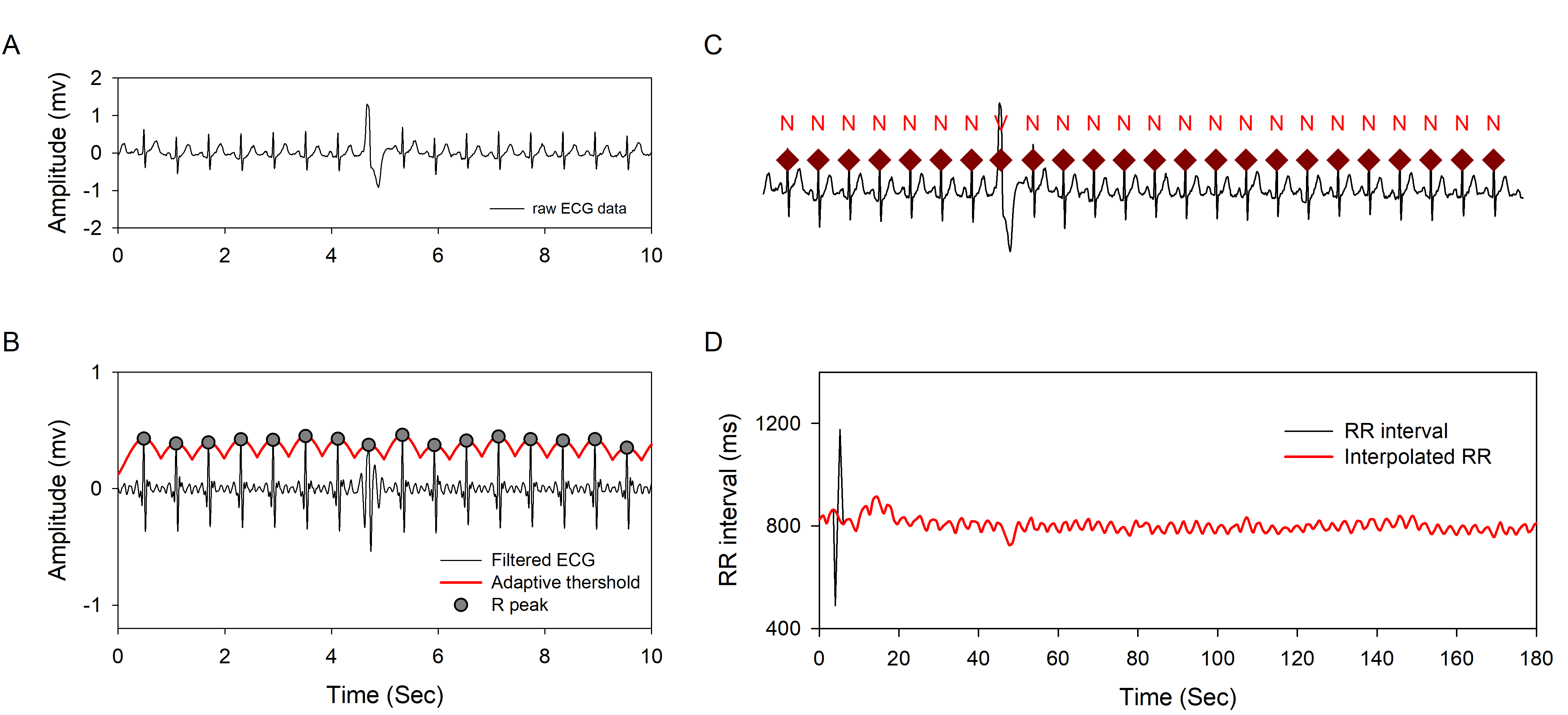


## Section 2: Continuous wavelet transform

The adopted continuous Morlet wavelet function is shown as

$$\Psi_{0}\left( \frac{t}{s} \right)=\pi^{-\frac{1}{4}}e^{j\omega_{0}t/s}e^{-1/2{(t/s)}^{2}}$$

where t and s are time and scale, respectively. W_0_ is the frequency of the wavelet. The convolution of the analyzed function g(t) with a scaled wavelet function shifted by $\tau$ is then defined as CWT.

$$W\left( s,\tau\right)=\int g\left( t \right)\Psi_{s}\left( t-\tau\right)dt$$

The relationship between the temporal and spectral aspects of the Morlet wavelet is shown below

$$\lambda=\frac{1}{f}=\frac{4\pi s}{\omega_{0}+\sqrt{2+\omega_{0}^{2}}}$$

where λ is the Fourier wavelength, and g(t) is derived from the evenly resampled normal-to-normal time series of heartbeats (RR intervals). The time function of the instantaneous HF (HFi) and LF (LFi) components is derived as the integral of CWT over the frequency band of HF (0.15–0.4 Hz) and LF (0.04–0.15 Hz), respectively. That is:

$$HFi(\tau)=\int_{0.15Hz}^{0.4Hz} W\left( f,\tau\right)df$$

$$LFi(\tau)=\int_{0.04Hz}^{0.15Hz} W\left( f,\tau\right)df$$

The temporal LFi/HFi ratio is the ratio of the LFi component to the HFi component. The instantaneous HF power (HFi) of the heartbeat series was used as a marker of vagal modulation^2^, while the ratio of the instantaneous LF power (LFi) to the HFi (LFi/HFi) was generally considered to be an indicator of the balance between sympathetic and vagal modulation.^3,4^

### Data normalization

To eliminate inter-subject differences in baseline autonomic function, the derived parameters were normalized individually to a common scale of 0–100 by their maximum and minimum values (formula 1):

$X^{'}=\frac{X-X_{min}}{X_{max}-X_{min}}\times100$(1)

where x is the value in the series, x_min_ is the lowest value, and x_max_ is the highest value. The transformed variables had the same normalized value range and distribution in each patient with the same normalized unit. The parameters were calculated and normalized offline using MATLAB (2016b, MathWorks Inc., Natick, MA, USA).

## Section 3: Fourier analysis

A Fourier spectrum power over the HF and LF frequency bands was calculated based on the published guidelines.^5^ A Gaussian window function centered at a certain time period was multiplied by the whole R-R signal before the Fourier transforms to maintain the same frequency resolution over the spectrum.

## Section 4: Pulse photoplethysmography analysis and amplitude extraction

To extract the pulse photoplethysmography (PPG) amplitude from the photoplethysmography, we applied a bandpass filter (e.g., 1–4 Hz) to remove noise, baseline wandering, and amplitude fluctuations related to respiration. The critical points of each PPG waveform were derived from the points of the zero-crossing of their first derivatives. Supplemental Figure S1 shows (A) the changes in finger PPG after administration of propofol and (B) the corresponding normalized PPG amplitude (B).


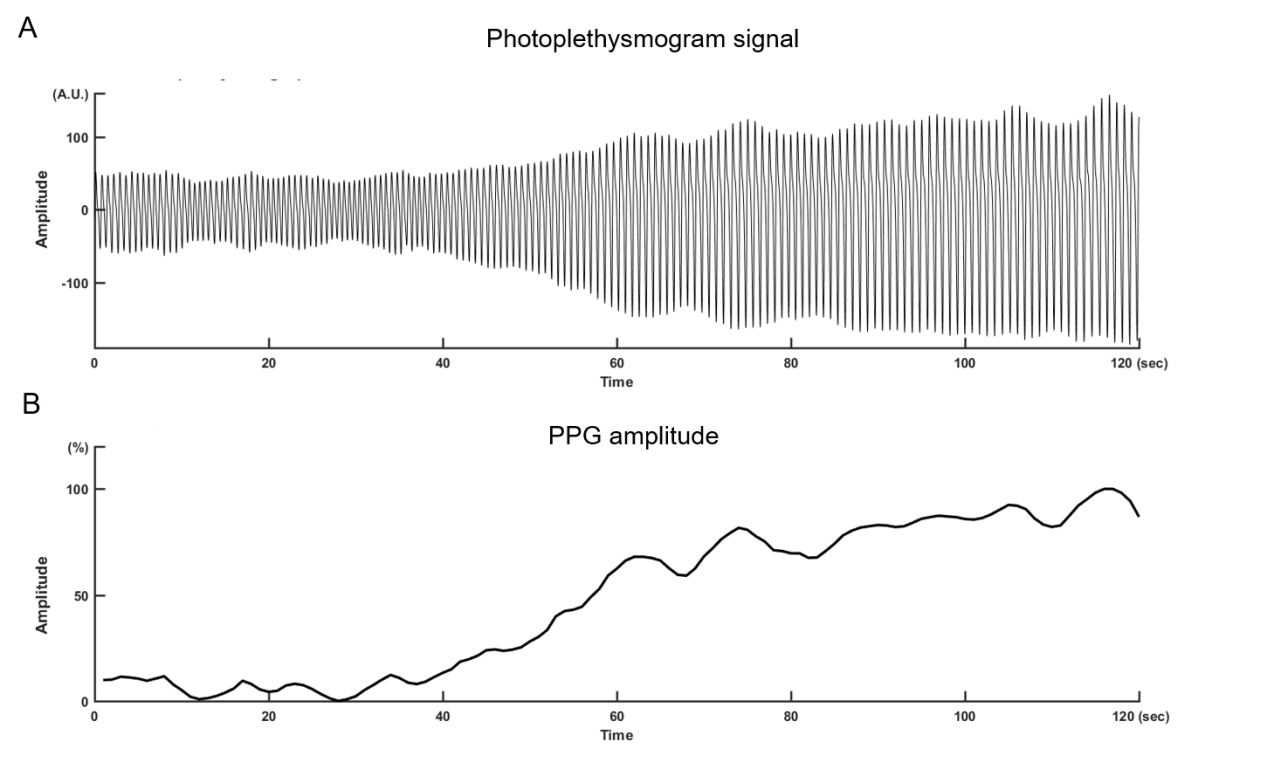


**Supplemental Figure S1.** (A) Changes in finger PPG after administration of propofol. (B) Corresponding normalized PPG amplitude. The amplitude between the trough and peak of each pulse wave in photoplethysmography amplitude (PPGA) was then calculated to assess changes in the state of the autonomic system, especially for peripheral vasoconstriction related to sympathetic activation. The PPGA sequences were also resampled at 1 Hz to match the temporal changes in autonomic function assessed by CWT.

# References

1. Lin C, Yeh CH, Wang CY, Shi W, Serafico BMF, Wang CH, Juan CH, Young HV, Lin YJ, Yeh HM, Lo MT: Robust Fetal Heart Beat Detection via R-peak Intervals Distribution. IEEE Trans Biomed Eng (2019).

2. Pomeranz B, Macaulay RJ, Caudill MA, Kutz I, Adam D, Gordon D, Kilborn KM, Barger AC, Shannon DC, Cohen RJ, et al.: Assessment of autonomic function in humans by heart rate spectral analysis. Am J Physiol (1985) 248:H151-3.

3. Malliani A, Pagani M, Lombardi F, Cerutti S: Cardiovascular neural regulation explored in the frequency domain. Circulation (1991) 84:482-92.

4. Montano N, Ruscone TG, Porta A, Lombardi F, Pagani M, Malliani A: Power spectrum analysis of heart rate variability to assess the changes in sympathovagal balance during graded orthostatic tilt. Circulation (1994) 90:1826-31.

5. Heart rate variability: standards of measurement, physiological interpretation and clinical use. Task Force of the European Society of Cardiology and the North American Society of Pacing and Electrophysiology. Circulation (1996) 93:1043-65.
